# Resolving the Taxonomic Enigma of *Adonis* in Eastern North China: An Integrated Phylogenomic Approach

**DOI:** 10.1002/ece3.73266

**Published:** 2026-03-19

**Authors:** Wen‐He Li, Ming‐Yang Li, Bo‐Wen Liu, Jian He, Jia‐Min Xiao, Le‐Le Lin, Ce Shang, Huanyu Wu, Shuang Qin, Zi‐Yi Li, Jin Cheng, Lei Xie

**Affiliations:** ^1^ State Key Laboratory of Efficient Production of Forest Resources Beijing Forestry University Beijing China; ^2^ Ecology and Nature Conservation Institute Chinese Academy of Forestry Beijing China

**Keywords:** Adonideae, phylogenomics, plastome, Ranunculaceae, RNA‐seq, taxonomy

## Abstract

The taxonomic identity of *Adonis* populations in eastern North China has long been controversial. Previous studies, relying on limited morphological data, have variably identified the Fuping (Hebei Province) population as *A. ramosa* , *A. amurensis* , or a subspecies of *A. ramosa* . To resolve this enigma, we conducted extensive field surveys and applied an integrated phylogenomic approach, utilizing genome skimming and RNA‐seq data from 44 samples representing 10 *Adonis* species in China. Our analyses, based on both complete plastid genomes and nuclear single‐copy orthologous genes, revealed that the Fuping population represents a distinct evolutionary lineage, which we describe as the new species *A. fupingensis*. In contrast, other populations from Beijing, Shandong, Henan, and Jiangsu were confirmed to belong to *A. ramosa* . We detected widespread gene tree discordance, primarily explained by incomplete lineage sorting (ILS), but also identified a clear case of ancient hybridization in the origin of *A. sutchuenensis*, with *A. brevistyla* and *A. fupingensis* as its parental donors. Despite the overall morphological similarity between *A. fupingensis* and *A. ramosa* , stable yet subtle differences in sepal and petal shape, root system architecture, and flowering phenology support their species‐level distinction. This study highlights the critical need for phylogenomic data to disentangle complex evolutionary histories and clarify taxonomic disputes in morphologically challenging plant groups.

## Graphical abstract

In this study, we demonstrate for the first time that Adonis species from Hebei Province, China are distinct species from *A. ramosa* , supported by robust phylogenomic analyses (plastid genome data and single copy nuclear genes) and morphological evidence. We reveal a complicated evolutionary history of Adonis species in China. The confirmation of Adonis fupingensis at the species level further necessitates its urgent conservation.

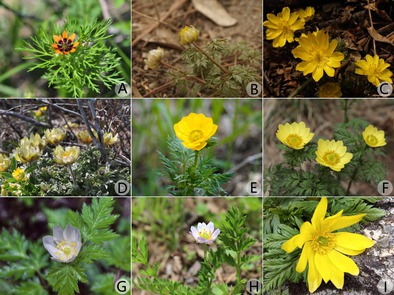

## 1 Introduction

Plant taxonomy, a cornerstone discipline of biological sciences dedicated to the exploration, circumscription, classification, and nomenclature of plant diversity, has experienced a fundamental paradigm shift over the past three decades (APG 1998; Li et al. 2024; PPG I 2016; Yang et al. 2022). This transformation was catalyzed by the advent of molecular systematics, which established a phylogenetic framework, thereby supplanting classifications based primarily on morphological similarity. This approach resolved previously intractable evolutionary relationships and rectified long‐standing taxonomic inaccuracies, redefining the architecture of the Plant Tree of Life across all taxonomic ranks (Qiu and Palmer 1999; Savolainen and Chase 2003). In recent years, phylogenomics has propelled the field into a period of accelerated discovery and deeper insight. The integration of genome‐scale data has dramatically increased the resolution of phylogenetic inferences, allowing for the precise delimitation of species and the elucidation of complex evolutionary histories that were once precluded by the traditional markers (One Thousand Plant Transcriptomes Initiative 2019; Kron et al. 2002; Li et al. 2023; Zhao et al. 2024; Duan et al. 2024). The phylogenomic method not only consolidates the systematic framework established by earlier molecular studies but also actively refines it, leading to a more robust, stable, and predictive classification system that truly reflects plant evolutionary history (Guo et al. 2023).

The genus *Adonis* L., belonging to the Ranunculaceae family, is well‐known for its species of perennial and annual herbaceous plants commonly referred to as the *Adonis* flowers. This genus is characterized by its distinct morphological features such as finely divided leaves, imbricate sepals, petals without a nectary, and spherical or ovoid aggregate achenes. There are about 30 species of *Adonis* that are widely distributed in cold and temperate areas of Eurasia and northern Africa (Tamura 1991; W. T. Wang 1994a, 1994b). The economic importance of *Adonis* flowers lies primarily in their medicinal properties and ornamental value. Many species within the genus are used in traditional medicine due to their bioactive compounds. For instance, the roots and whole plants of certain *Adonis* species contain cardiotonic glycosides like Adoniside, which have been used to treat heart conditions such as congestive heart failure and palpitations (Shang et al. 2019). The plants are also known for their diuretic and sedative effects, contributing to their use in treating a variety of ailments (Fu and Zhang 1995). Although *Adonis* comprises a relatively small number of species in the family, its morphological classification presents significant challenges.

*Adonis* has been most comprehensively revised by W. T. Wang (1994a, 1994b). His classification organized the genus into two distinct subgenera based on life history: the annual subg. *Adonis* and the perennial subg. *Adonanthe* (Spach) W. T. Wang, with each further divided into three sections. However, at species level, many closely related species within *Adonis* exhibit remarkably similar vegetative morphology, leading to significant identification difficulties, especially when diagnostic floral characters are unavailable in herbarium specimens. These taxonomic complexities have been particularly noted in Wang's classification (W. T. Wang (1994a, 1994b)), where overlapping morphological characters among closely related species frequently complicate accurate specimen determination. The situation underscores the need for complementary approaches, such as molecular phylogenetic study, to clarify species boundaries in this challenging genus (Vasques et al. 2025).

The genus *Adonis* in China comprises approximately 10 species, among which *A. aestivalis* L. is the only annual species while the remaining nine are perennial (Fu and Robinson 2001). Historically, the distribution of *Adonis* in China was documented primarily in northeastern provinces, as well as northern regions including Inner Mongolia, Gansu, and Xinjiang, along with mountainous areas of western and southwestern China such as Qinghai, Hubei, Sichuan, Yunnan, and Xizang (W. T. Wang 1994a, 1994b; Fu and Robinson 2001). However, records from eastern North China (Beijing, Hebei, Shandong, Henan, and Jiangsu) are notably scarce, and the taxonomic status of the limited existing specimens remains controversial (W. T. Wang 1994a, 1994b).

W. T. Wang (1994a, 1994b) classified the *Adonis* populations from Fuping County, Hebei Province as *A. ramosa* subsp. *fupingensis* W. T. Wang. In the same revision, he refrained from assigning a definitive species to the Shandong specimen due to the presence of fruits but absence of flowers. In contrast, other taxonomists have alternatively identified the Fuping specimen as *A. amurensis* Regel & Radde (J. W. Wang 1986) or synonymized *A. ramosa* subsp. *fupingensis* under *A. ramosa* Franch. (Fu and Robinson 2001).

Recent observations by botanists and plant enthusiasts have expanded the known range of *Adonis* in eastern North China, with new reports from Beijing, Henan, and northern Jiangsu provinces. Current evidence indicates that the genus *Adonis* exhibits a sparse and fragmented distribution in eastern North China, with scattered records in Huairou (Beijing), Fuping (Hebei), Rushan (Shandong), Huixian (Henan), and as far south as Lianyungang (Jiangsu). However, the extreme scarcity of specimens hinders its taxonomic studies, and the precise species identification of these populations still awaits comprehensive analyses.

Molecular phylogenetic investigations have markedly enhanced our comprehension of the phylogenetic relationships within the genus *Adonis* (Son et al. 2016; Karahan et al. 2022; Ling et al. 2025; Vasques et al. 2025). Notably, the comprehensive study conducted by Ling et al. (2025) achieved substantial progress in elucidating the phylogeny of the genus through the reconstruction of its evolutionary history using ITS sequences and eight plastid DNA markers, incorporating the most extensive taxonomic sampling to date. Their phylogenetic analyses provided robust support for the monophyly of *Adonis* at the genus level, while also confirming the distinct evolutionary lineages corresponding to the two established subgenera: the annual subg. *Adonis* and the perennial subg. *Adonanthe*. However, this study did not include representatives of *Adonis* populations from eastern North China. Although the research provides valuable insights into the genus‐wide phylogeny, the absence of these eastern North China samples leaves open questions about the phylogenetic placement of these *Adonis* populations and their potential influence on the infrageneric classification of the genus.

Recent studies have demonstrated that the limited phylogenetic information provided by Sanger sequencing data often proves insufficient for resolving species boundaries among closely related taxa within some Ranunculaceae taxa (He et al. 2021; Lyu et al. 2023). With the advancement of next‐generation sequencing (NGS) technologies and the continuous reduction in sequencing costs, genomic data have become the key resources for resolving plant phylogenetic relationships and revising taxonomic frameworks (Jiao et al. 2023; Chen et al. 2023). The synergistic use of organellar and nuclear genome data not only strengthens phylogenetic inference but also reveals previously undetectable evolutionary history, such as hybridization and introgression events (Zhang et al. 2020), revolutionizing our understanding of species boundaries.

In this study, we conducted comprehensive field surveys and collections of *Adonis* populations across eastern North China, supplemented by extensive sampling of *Adonis* species distributed throughout China. Utilizing genome skimming and RNA‐seq approaches, we obtained complete plastome sequences and nuclear single‐copy orthologous genes for phylogenetic reconstruction. This study aims to: (1) clarify species delimitation of *Adonis* in eastern North China, (2) resolve their phylogenetic relationships with other perennial species, and (3) establish a taxonomic framework for understanding the genus's diversity in this area and informing conservation strategies.

## 2 Materials and Methods

### 2.1 Plant Sampling

**FIGURE 1:**
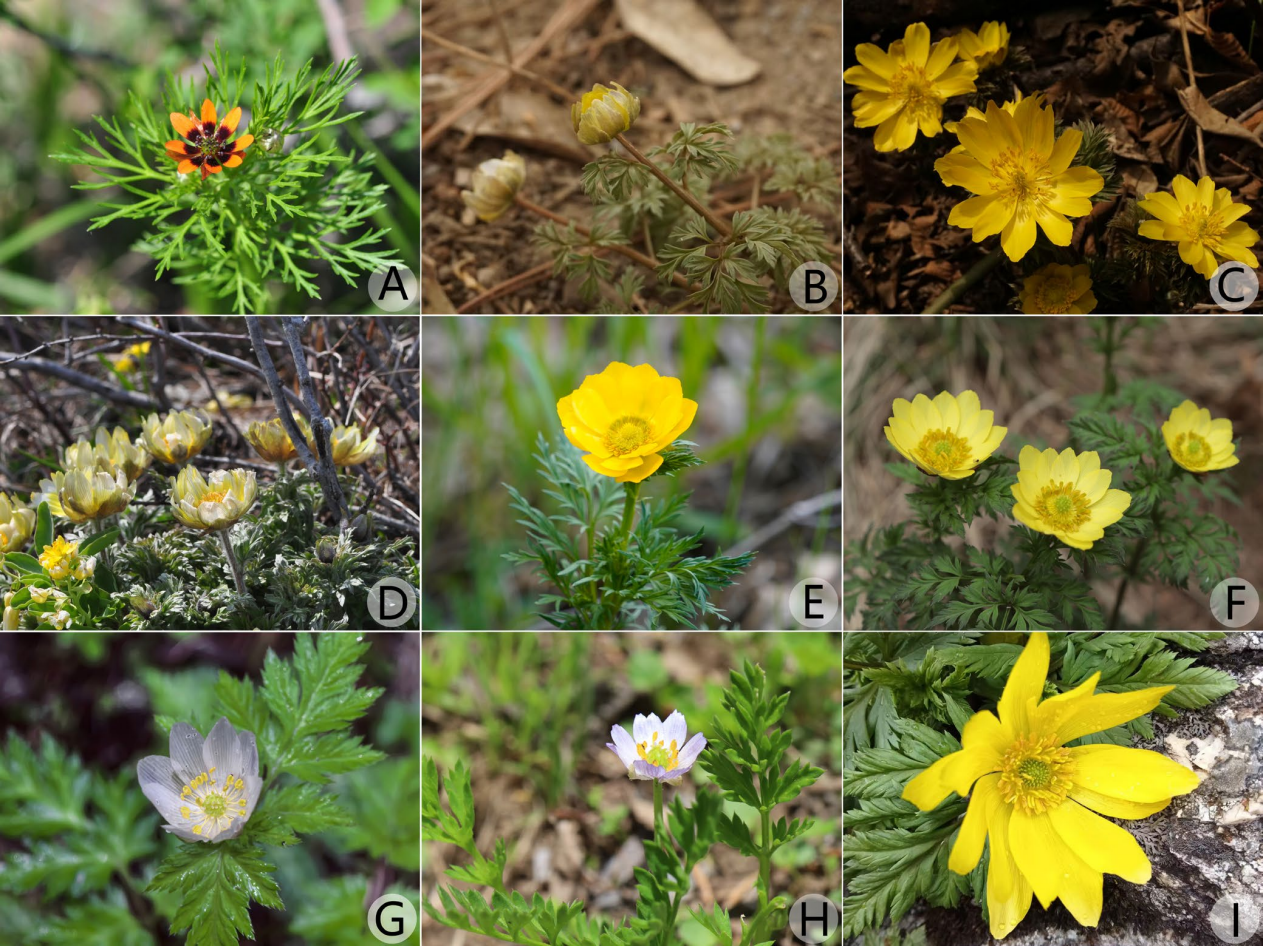
Field photographs of *Adonis* plants. (A) *A. aestivalis* var. *parviflora* (photographed by Shao‐Yu Guo, in Altay, Xinjiang province); (B) *A. amurensis* (photographed by Wen‐He Li, Cultivated in Chengnan Park, Huairou district, Beijing); (C) *A. ramosa* (photographed by Wen‐He Li, in Huixian county, Henan province); (D) *A. villosa* (photographed by Shao‐Yu Guo, in Altay, Xinjiang province); (E) *A. sibirica* (photographed by Le‐Le Lin, in Altay, Xinjiang province); (F) *A. fupingensis* (photographed by Jia‐Min Xiao, in Fuping county, Hebei province); (G) *A. brevistyla* (photographed by Wen‐He Li, in Bomi county, Xizang province); (H) *A. coerulea* (photographed by Jia‐Min Xiao, in Jianzha county, Qinghai province); (I) *A. sutchuenensis* (photographed by Lian‐Jie Li, in Taibai county, Shaanxi province).

In this study, we conducted extensive field surveys and collected leaf samples of *Adonis* distributed in Beijing, Hebei, Henan, Shandong, and Jiangsu provinces. Multiple individuals from each site are used for phylogenomic analysis. Other *Adonis* species from north‐eastern, north‐western, and south‐western China were collected from the field (Figure 1) for phylogenetic reconstruction. In total, 10 species and 41 samples of *Adonis* from China are included in this study. Six species of *Trollius* L. and one species of *Calathodes* Hook. f. & Thoms., which were shown by previous phylogenetic studies to be closely related to *Adonis* (Wang et al. 2010; Zhai et al. 2019), were also collected from the field and chosen as the outgroups (Table 1). Voucher specimens deposited in the Herbarium of Beijing Forestry University (BJFC, herbarium code follows Thiers 2017) were identified by the last author according to the revisions by W. T. Wang (1994a, 1994b) and Fu and Robinson (2001).

**TABLE 1:** Information of samples used in this study.

Species	Locality	Voucher	Herbarium	Plastid genome	RNA‐seq
*Calathodes oxycarpa*	Emei, Sichuan, China	L. Xie, 2024050902‐8	BJFC	Y	Y
*Trollius altaicus*	Bortala, Xinjiang, China	LL. Lin, 20240618009	BJFC	Y	Y
*Trollius chinensis*	Zhuolu, Hebei, China	L. Xie, 2023061310	BJFC	Y	Y
*Trollius pumilus*	Dingqing, Xizang, China	L. Xie, 2023080915	BJFC	Y	Y
*Trollius ranunculoides*	Xunhua, Qinghai, China	L. Xie, 2024060502	BJFC	Y	Y
*Trollius lilacinus*	Fukang, Xinjiang, China	LL. Lin, 20240611005	BJFC	Y	Y
*Trollius yunnanensis*	Jiangda, Xizang, China	L. Xie, 2023081015‐1	BJFC	Y	Y
*Adonis aestivalis* var. *parviflora*	Altay, Xinjiang, China	LL. Lin, I‐4422	BJFC	Y	Y
*Adonis amurensis*	Tonghua, Jilin, China	JL. Yu, 2024050305‐1	BJFC	Y	Y
*Adonis amurensis*	Tonghua, Jilin, China	JL. Yu, 2024050405‐1	BJFC	Y	Y
*Adonis amurensis*	Tonghua, Jilin, China	JL. Yu, 2024050405‐3	BJFC	Y	Y
*Adonis amurensis*	Cult. in Beijing, Chengnan Park	WH. Li, 2025031401	BJFC	Y	Y
*Adonis amurensis*	Japan (Cult. in Beijing)	WH. Li, WHJP01	BJFC	Y	Y
*Adonis bobroviana*	Xining, Qinghai, China	WH. Li, 2024060601‐1	BJFC	Y	Y
*Adonis bobroviana*	Xining, Qinghai, China	WH. Li, 2024060601‐2	BJFC	Y	Y
*Adonis bobroviana*	Xining, Qinghai, China	WH. Li, 2024060601‐3	BJFC	Y	Y
*Adonis coerulea*	Jianzha, Qinghai, China	JM. Xiao, 2024060508‐1	BJFC	Y	Y
*Adonis coerulea*	Jianzha, Qinghai, China	JM. Xiao, 2024060508‐2	BJFC	Y	Y
*Adonis coerulea*	Jianzha, Qinghai, China	JM. Xiao, 2024060508‐3	BJFC	Y	Y
*Adonis brevistyla*	Bomi, Xizang, China	WH. Li, 2025053006‐1	BJFC	Y	Y
*Adonis brevistyla*	Bomi, Xizang, China	WH. Li, 2025053006‐9	BJFC	N	Y
*Adonis brevistyla*	Bomi, Xizang, China	WH. Li, 2025053006‐12	BJFC	N	Y
*Adonis brevistyla*	Bomi, Xizang, China	WH. Li, 2025053006‐16	BJFC	Y	N
*Adonis brevistyla*	Bomi, Xizang, China	WH. Li, 2025053006‐18	BJFC	Y	N
*Adonis fupingensis*	Fuping, Hebei, China	WH. Li, 2023051702	BJFC	Y	Y
*Adonis fupingensis*	Fuping, Hebei, China	WH. Li, 2023051704	BJFC	Y	Y
*Adonis fupingensis*	Fuping, Hebei, China	WH. Li, 2023051705	BJFC	Y	Y
*Adonis ramosa*	Lianyungang, Jiangsu, China	WH. Li, 2025032301‐1	BJFC	Y	Y
*Adonis ramosa*	Lianyungang, Jiangsu, China	WH. Li, 2025032303‐1	BJFC	Y	Y
*Adonis ramosa*	Xinxiang, Henan, China	L. Xie, 2025031201‐4	BJFC	Y	Y
*Adonis ramosa*	Xinxiang, Henan, China	L. Xie, 2025031201‐5	BJFC	Y	Y
*Adonis ramosa*	Xinxiang, Henan, China	L. Xie, 2025031201‐17	BJFC	Y	Y
*Adonis ramosa*	Huairou, Beijing, China	L. Xie, 2025032802‐3	BJFC	Y	Y
*Adonis ramosa*	Huairou, Beijing, China	L. Xie, 2024030801	BJFC	Y	Y
*Adonis ramosa*	Tonghua, Jilin, China	JL. Yu, 2024050304‐1	BJFC	Y	Y
*Adonis ramosa*	Tonghua, Jilin, China	JL. Yu, 2024050304‐2	BJFC	Y	Y
*Adonis ramosa*	Tonghua, Jilin, China	JL. Yu, 2024050304‐5	BJFC	Y	Y
*Adonis ramosa*	Rushan, Shandong, China	L. Xie, 2025041001‐1	BJFC	Y	Y
*Adonis ramosa*	Rushan, Shandong, China	L. Xie, 2025041001‐5	BJFC	Y	Y
*Adonis ramosa*	Rushan, Shandong, China	L. Xie, 2025041001‐9	BJFC	Y	Y
*Adonis sibirica*	Altay, Xinjiang, China	LL. Lin, 20250527001	BJFC	Y	Y
*Adonis sibirica*	Burqin, Xinjiang, China	C. Shang, I‐4786	BJFC	Y	Y
*Adonis sibirica*	Burqin, Xinjiang, China	C. Shang, I‐4749	BJFC	Y	Y
*Adonis sutchuenensis*	Yichang, Hubei, China	WH. Li, WH25032502‐1	BJFC	Y	Y
*Adonis sutchuenensis*	Yichang, Hubei, China	WH. Li, WH25032502‐2	BJFC	Y	Y
*Adonis villosa*	Altay, Xinjiang, China	LL. Lin, 20240520011	BJFC	Y	Y
*Adonis villosa*	Altay, Xinjiang, China	C. Shang, I‐4247	BJFC	Y	Y
*Adonis villosa*	Altay, Xinjiang, China	J. He, 20180525‐01	BJFC	N	Y

Although the sampling for the phylogenomic analysis in this study covered most species distributed in China, some *Adonis* species from Xinjiang [*A. chrysocyatha* Hook. f. et Thoms. and *A. tianschania* (Adolf) Lipsch.], Central and Northern Asia ( *A. mongolica* Simon., *A. turkestanica* (Korsh.) Adolf, *A. volgensis* Stev.), and the Himalayas ( *A. nepalensis* Simon.) were not included. This gap in sampling may affect the inference of the systematic position of the Fuping population. Therefore, we downloaded all Sanger sequencing data from Ling et al. (2025), whose sampling encompassed the entire genus, and combined it with the chloroplast (*mat*K, *rbc*L, *atp*B‐*rbc*L, *rps*16, *trn*G, *trn*H‐*psb*A, *trn*L‐F, *trn*S‐*trn*G) and nrITS sequences assembled from our genome skimming data for a preliminary analysis. All sequence alignment and phylogenetic tree construction procedures strictly followed Ling et al. (2025).

### 2.2 Genome Skimming Sequencing

The extraction of total genomic DNA from silica‐dried leaves was performed at Berry Genomics Co. Ltd. (https://www.bioon.com.cn/company/index/df7a2b444074) using a genomic DNA extraction kit following manufacturer instructions (Tiangen Biotech Co., Beijing, China). The DNBSEQ‐T7 library inc'orporated 1 μg of DNA from each sample as the initial input, constructing the sequencing libraries in accordance with the guidelines provided by theVAHTS Universal DNA Library Prep Kit for MGI (Vazyme, Nanjing, China). Then, 2 × 150 bp paired‐end libraries were sequenced using a DNBSEQ‐T7 sequencer (BGI, Shenzhen, China). All the sequenced samples yielded around 10 Gbp of raw data for plastid genome assembling (Table A1).

### 2.3 RNA Extraction and Sequencing

We followed the pipeline of He et al. (2022) to obtain the RNA sequence from silica gel‐dried tissues. We sent our field collected leaf tissues to Berry Genomics Co. Ltd. (https://www.bioon.com.cn/company/index/df7a2b444074) for RNA extraction, library construction, and next‐generation sequencing. A total of 100 mg of leaf tissue was ground for each sample, and total RNA was subsequently extracted utilizing a standard TRIzol‐based RNA extraction kit (TRIzon, CoWin Biosciences, Jiangsu, PR China), in line with the manufacturer's protocol. RNA integrity numbers (RIN) and the ratios of 26S to 18S ribosomal RNA (rRNA) were analyzed with the aid of a 2100 Bioanalyzer (Agilent Technologies, Santa Clara, California, USA). Subsequently, the cDNA library was constructed from the total RNA extracts via the method of reverse transcription PCR. The libraries were processed on the NovaSeq 6000 platform (Illumina, San Diego, California, USA), which ultimately produced nearly 6 Gb of raw data for each sample. The complete set of raw reads has been uploaded in the CNCB National Genomics Data Center under BioProject: PRJCA050622 (Table A2). Raw data quality was initially assessed and visualized using the FASTX‐Toolkit (http://hannonlab.cshl.edu/fastx_toolkit/). Subsequently, data cleaning and preprocessing were performed using Trimmomatic v.0.39 (Bolger et al. 2014) to remove adapter sequences and filter out low‐quality reads (e.g., sliding window trimming and minimum length filtering), thereby ensuring the acquisition of high‐quality, clean data for downstream analyses.

### 2.4 Plastome Sequence Assembling

The complete plastome was assembled *de novo* from genome skimming data utilizing GetOrganelle (Jin et al. 2020) with standard settings, or alternatively, it was constructed using Geneious Prime (Kearse et al. 2012) in accordance with our prior studies (He et al. 2019, 2021) when GetOrganelle was unable to produce a complete plastome assembly. We used 20 iterations of Fine Tuning in Geneious Prime (Kearse et al. 2012) to connect the gaps present between contigs. Annotations were carried out through the Plastid Genome Annotator (PGA, Qu et al. 2019), and a subsequent manual verification was performed with Geneious Prime (Kearse et al. 2012). All sequences that have been annotated are available in the NCBI database (Table A1).

### 2.5 Nuclear Genomic Data Assembling

The raw sequencing reads from each sample were assembled into contigs using Trinity v2.14.5 (Grabherr et al. 2011) with default settings. To retain the longest representative transcript per gene, we applied the Trinity‐included script “get_longest_isoform_seq_per_trinity_gene.pl”. Redundant contigs were then filtered using CD‐HIT (Li and Godzik 2006) with the “cd‐hit‐est” command under default parameters. For downstream orthologous and phylogenetic analyses, we predicted open reading frames (ORFs) and coding sequences (CDSs) from the assembled transcripts using TransDecoder v3.0.1 (Haas et al. 2013; available at https://github.com/TransDecoder/TransDecoder). Finally, transcriptome completeness was assessed by aligning against the BUSCO database (Simão et al. 2015) to evaluate the presence of conserved single‐copy orthologs.

### 2.6 Identification, Filtering, and Alignment of Nuclear Orthologous Genes

To identify single‐copy orthologous genes (SCOGs), we performed an all‐versus‐all BLAST analysis on protein‐coding contigs from all 47 samples using Proteinortho v.6 (Lechner et al. 2011). SCOGs were extracted from the Proteinortho results using a custom Python script (available at GitHub https://github.com/Jhe1004/Get_SCOG_from_Proteinortho; He et al. 2022), retaining only those present in at least 32 samples (≥ 70% of the dataset). To exclude potential organellar contamination, we filtered the SCOGs by aligning them against the *Adonis* plastid genome (NC 072342.1) and the *Adonis annua* L. mitochondrial genome (OZ123192.1) via BLAST.

Orthology detection based on BLAST searches may produce unreliable results due to various factors including assembly errors, incomplete lineage sorting, or reading frame shifts (Yang and Smith 2014). These issues can lead to phylogenetic anomalies, particularly when sequences are incorrectly grouped with non‐orthologous counterparts, resulting in artificially elongated branches. To address this concern, we implemented Treeshrink v.1.3.9 (Mai and Mirarab 2017) to systematically identify and evaluate unusually long branches in individual gene trees, which helps detect potential alignment artifacts. Our gene tree construction pipeline involved: (1) multiple sequence alignment of single‐copy orthologs using MAFFT v.7.475 (Katoh and Standley 2013) with default settings. (2) removing columns with > 20% missing data by using another Python script (GitHub https://github.com/Jhe1004/DelMissingSite), following the approach of Duvall et al. (2020).(3) phylogenetic reconstruction with RAxML v.8.2.12 (Stamatakis 2014) employing the GTR+G evolutionary model. Following Treeshrink analysis, we pruned sequences exhibiting suspiciously long branches from the final alignments.

### 2.7 Phylogenetic Analysis

Prior to multiple sequence alignment of the plastome data, we performed gene order adjustments in *Adonis* using Geneious Prime (Kearse et al. 2012) to account for structural variations in the large single‐copy (LSC) regions between *Adonis* and outgroup taxa (He et al. 2019; Zhai et al. 2019). The reorganized sequences were subsequently aligned with MAFFT v.7.221 (Katoh and Standley 2013), followed by refinement using a custom Python script (available at https://github.com/Jhe1004/DelMissingSite) to eliminate poorly aligned regions, applying a 20% gap threshold as described by Duvall et al. (2020).

Phylogenetic analyses were conducted on the complete plastid genome data separately using maximum likelihood (ML) and Bayesian inference (BI) approaches. Maximum likelihood phylogenetic reconstruction was conducted using RAxML v.8.2.12 (Stamatakis 2014) with the GTR + G substitution model and 1000 bootstrap pseudo‐replicates for node support estimation. The Bayesian inference (BI) analysis was conducted in ExaBayes (Aberer et al. 2014) under the same substitution model as used for the ML analysis. Each run comprised two independent Markov chain Monte Carlo (MCMC) simulations, with each simulation operating four chains (three heated and one cold) for 2 million generations, sampling trees every 100 generations. The first 25% of sampled trees from each run were discarded as burn‐in. The post‐burn‐in trees were then used to construct a majority‐rule consensus tree and estimate posterior probabilities. Convergence was confirmed by examining trace plots and verifying that the Effective Sample Size (ESS) for all parameters was greater than 200. The final sequence alignments are available in the Zenodo repository (DOI: https://doi.org/10.5281/zenodo.16964931).

Regarding the nuclear data, we selected two SCOGs datasets with alignment length of at least 1000 bp (SCOG1000) and 2000 bp (SCOG2000) for phylogenetic analysis, to avoid potential inaccuracies in gene tree estimation stemming from limited sequence length (Xiao et al. 2022). We employed two complementary approaches: concatenation‐based and coalescent‐based methods for analyzing the two SCOG data sets. In the concatenation approach, we combined all SCOG alignments of each data set into a single supermatrix using the sequence concatenation function in Geneious Prime (Kearse et al. 2012). This alignment was subsequently subjected to maximum likelihood analysis through the CIPRES Science Gateway v.3.3 platform (Miller et al. 2012), utilizing RAxML v.8.2.12 (Stamatakis 2014) with the GTR+G evolutionary model and 500 bootstrap iterations to assess nodal support. Coalescent‐based phylogenetic reconstruction was performed using ASTRAL v.5.6.3 (Zhang et al. 2018) following a two‐step summary approach. Individual gene trees were generated with RAxML v.8.2.12 (Stamatakis 2014) under the GTR+G model, incorporating 500 bootstrap replicates per tree.

To supplement our phylogenomic sampling and place the Fuping population within a genus‐wide context, we integrated our genome‐skimming sequences (*mat*K, *rbc*L, *atp*B‐*rbc*L, *rps*16, *trn*G, *trn*H‐*psb*A, *trn*L‐F, *trn*S‐*trn*G, and nrITS) with the comprehensive Sanger sequencing dataset from Ling et al. (2025). While sequence alignment followed the protocols of Ling et al. (2025), phylogenetic reconstruction was performed using the same Maximum Likelihood (ML) and Bayesian Inference (BI) approaches as described for our plastid genome data. Given the well‐documented cyto‐nuclear discordance in *Adonis*, chloroplast and nuclear datasets were analyzed separately to infer the approximate systematic position of the Fuping population. This preliminary placement provided a broader taxonomic framework for our subsequent high‐resolution analyses using the genomic datasets generated in this study.

### 2.8 Analysis of Tree Discordance

This study investigated phylogenetic incongruence among nuclear gene trees as well as between plastid and nuclear gene trees, assessing potential biological explanations. We systematically ruled out confounding factors such as sampling errors, stochastic variance, and paralogous gene effects (Zou and Ge 2008) and then evaluated the contributions of incomplete lineage sorting (ILS) and hybridization to gene tree discordance.

First, we assessed topological conflicts within nuclear gene trees (SCOG1000 dataset). To minimize stochastic error, only gene trees with average support values exceeding 60 were retained for downstream analyses. Using Phyparts v.0.0.1 (Smith et al. 2015), we compared individual nuclear gene trees against the species tree, quantifying the percentage of concordant gene trees at each node and visualizing results with pie charts. Additionally, to enhance resolution of single‐gene conflicts, we generated cloud tree plots via the Python package Toytree v.2.0.5 (Eaton 2020). Ultrametric gene trees were time calibrated with TreePL v.1.0 (Smith and O’Meara 2012) and, using the python package DendroPy v.4.5.2 (Sukumaran and Holder 2010), each gene tree's nodes were calibrated by age estimates from the recently published study (Ling et al. 2025). Ultimately, we obtained 71 ultrametric gene trees for cloud tree plots.

To determine whether ILS could account for nuclear gene tree conflicts, we applied a multispecies coalescent (MSC) model in a simulation‐based framework (Yang et al. 2020; Morales‐Briones et al. 2021). A strong fit between the coalescent model and empirical gene trees would suggest ILS as the primary driver of discordance. Using the “sim.coaltree.sp” function in Phybase v.1.5 (Liu and Yu 2010), we simulated 10,000 gene trees under the MSC model, with the input species tree derived from the SCOG1000 dataset. We then computed pairwise distances between empirical gene trees and the species tree using DendroPy v.4.5.2 (Sukumaran and Holder 2010), comparing their distribution against simulated gene trees via histogram visualization. Furthermore, we examined cyto‐nuclear discordance through coalescent simulations (Rose et al. 2021; He et al. 2025). Employing the same “sim.coaltree.sp” function (Phybase v.1.5), we generated another 10,000 simulated gene trees and compared them to the plastome phylogeny using PhyParts v.0.0.1 (Smith et al. 2015). Discordant nodes receiving substantial support from simulated trees would imply ILS as a plausible cause of phylogenetic conflict.

### 2.9 Hybridization Detection and Visualization

To further elucidate the reticulate evolution among *Adonis* species, this study adopts the method proposed by He et al. (2025) to detect hybridization events. Hybridization events were assessed through the application of the HyDe package (Blischak et al. 2018). For the subsequent synthesis and interpretation of these findings, particularly those pertaining to ancestral nodes, we used the “VisualHyDe” Python script (He et al. 2025), accessible at https://github.com/Jhe1004/VisualHyde, featuring a “Node Mode” designed to deduce ancient reticulation. To mitigate the incidence of false positives resulting from variations in lineage rates (Koppetsch et al. 2024; Pang and Zhang 2025), a rigorous filtering protocol, incorporating null‐hypothesis simulation, was executed consistent with He et al. (2025).

## 3 Results

### 3.1 Genome Skimming and Transcriptome Data

The genome skimming data size of the sequenced sample ranged from 9.75 Gbp ( *A. ramosa* 2025032301‐1) to 20.99 Gbp ( *A. amurensis* 2024050305‐1), and the Q20 was 98.44%–100.00%, Q30 was 95.28%–97.78% (Table A1). The data size of transcriptomes ranged from 6.00 Gbp ( *A. villosa* Ledeb.) to 11.87 Gbp (*A. fupingensis* 2023051705), and the Q20 was 97.40%–99.57%, Q30 was 92.80%–97.79%. After *de novo* assembly with Trinity and removal of redundant sequences using CD‐HIT, 14,337–29,679 transcripts were retained. The N50 length of these transcripts ranged from 738 bp to 1344 bp, and the BUSCO completeness assessment of the assemblies ranged from 33.1% ( *A. aestivalis* var. *parviflora* M. Bieb.) to 75.4% ( *A. brevistyla* Franch. 2025050306‐1) (Table A2).

### 3.2 Phylogenetic Position of the Fuping *Adonis* Using Sanger Sequencing Data

This study downloaded all eight chloroplast fragments and the nrITS fragment from the research of Ling et al. (2025). These were combined with the corresponding fragments assembled in this study using genome skimming methods for phylogenetic reconstruction (Figures A1 and A2). The overall topologies of the plastid and ITS phylogenetic trees are congruent with those reported by Ling et al. (2025) and are also largely consistent with the phylogenomic results obtained in this study, albeit with lower resolution and statistical support. Analyses of both datasets revealed that the annual subg. *Adonis* and the perennial subg. *Adonanthe* each form distinct clades.

On the plastid tree, the Fuping population clusters with part of the *A. brevistyla* Franch. and *A. sutchuenensis* Franch. samples (Figure A1). However, due to low statistical support, the interspecific relationships within its larger clade (including *A. davidii* Franch., *A brevistyla*, *A. sutchuenensis*, *A. apennina* L., *A. sibirica* Patrin ex Ledeb., *A. mongolica* and *A. coerulea* Maxim.) remain unresolved. In the nrITS tree (Figure A2), the Fuping population clusters with *A. sibirica* , *A. apennina*, and *A. mongolica* , albeit with very weak statistical support. Despite the overall low resolution of the phylogenetic trees constructed from Sanger sequencing data, they nonetheless suggest that the Fuping population is distantly related to species such as the Himalayan *A. nepalensis* and the Central Asian *A. tianschanica* and *A. turkestanica* .

### 3.3 Plastid Genome and Nuclear Single‐Copy Orthologous Groups (SCOGs)

We acquired a total of 38 *Adonis* plastome sequences (45 sequences including outgroups, Table A1) ranging from 153,282 bp ( *A. aestivalis* var. *parviflora*) to 160,296 bp (*Calathodes oxycarpa* Sprag.). The number and arrangement of the plastid genes of all the *Adonis* species are identical, all contained a pair of IRs (26,048–26,624 bp.) separated by a large single copy region (83,386–88,311 bp) and a small single copy region (17,764–18,530 bp). All plastomes encoded a set of 112–114 genes, including 78–80 protein‐coding genes, 30 transfer RNAs and four ribosomal RNAs (Table A1). After removing IRa and poor alignment region, we finally obtained a matrix with aligned length of 129,718 bp for phylogenetic analysis.

For the transcriptome data, we successfully obtained RNA reads from 39 *Adonis* samples (46 samples including outgroups). Following the removal of 46 organelle genes, we identified 7003 one‐to‐one single‐copy orthologous genes (SCOGs) through homologous clustering. To evaluate the phylogenetic signal of individual genes, we calculated the Average Bootstrap Support (ABS) for each gene tree, defined as the mean bootstrap value across all internal nodes of the Maximum Likelihood tree. We further filtered the dataset by excluding 3518 genes shorter than 1000 bp. Consequently, the SCOG1000 dataset comprised 3485 genes (including a subset of 2242 genes with an ABS > 60), while the SCOG2000 dataset (genes > 2000 bp) contained 706 genes.

### 3.4 Phylogenetic Inference

**FIGURE 2:**
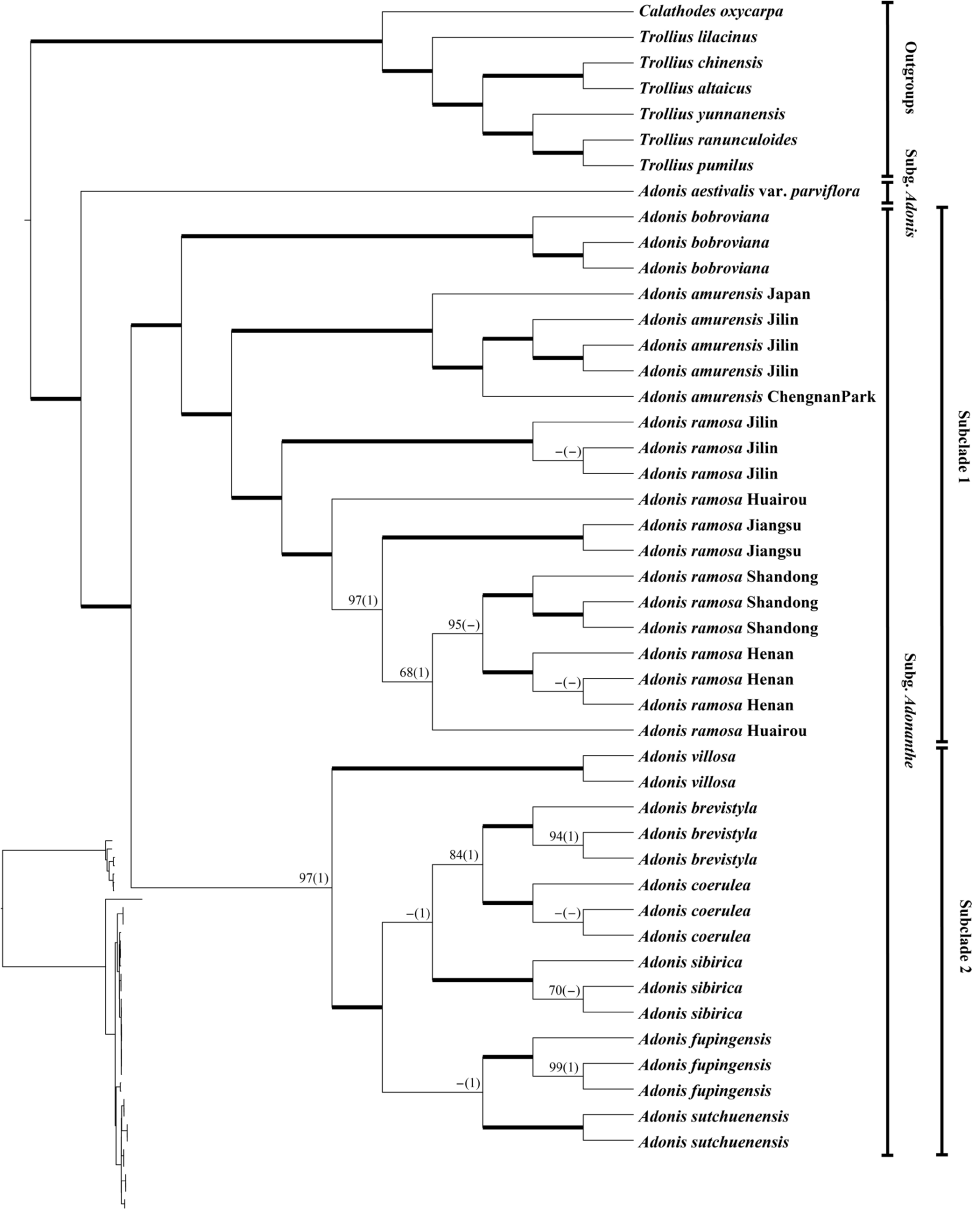
Phylogenies of *Adonis* species, inferred from the complete plastid genome sequences using the maximum likelihood (ML) method. Phylogram of the ML tree is shown left below. ML bootstrap values (> 50) and posterior probability (PP) values (> 0.95) of Bayesian Inference are shown at each node. Internal branches, which are fully supported by both analyses, are in bold. ML bootstrap values < 50 and PP values < 0.95 are shown as ‐.

For the plastid genome data, except some terminal clades with relatively weak support values, the majority of branches received full or strong support (Figure 2). The annual subg. *Adonis* and the perennial subg. *Adonanthe* were clearly separated. Within the subg. *Adonis* clade, two subclades were resolved. One (subclade 1) contains *Adonis* populations from Beijing, Shandong, Henan, and Jiangsu provinces together with other species from north‐eastern and north‐western china (including, *A. ramosa* , *A. amurensis* , and *A. bobroviana* Simonov), whereas the other one (subclade 2) contains *Adonis* population from Hebei Fuping and other species from western and south‐western China (including *A. villosa* , *A. brevistyla* , *A. coerulea* Maxim., *A. sutchuenensis* Franch., and *A. sibirica* Patrin ex Ledeb.). Population from Hebei Fuping is sister to *A. sutchuenensis*, and other populations from eastern North China are nested within *A. ramosa* clade.

**FIGURE 3:**
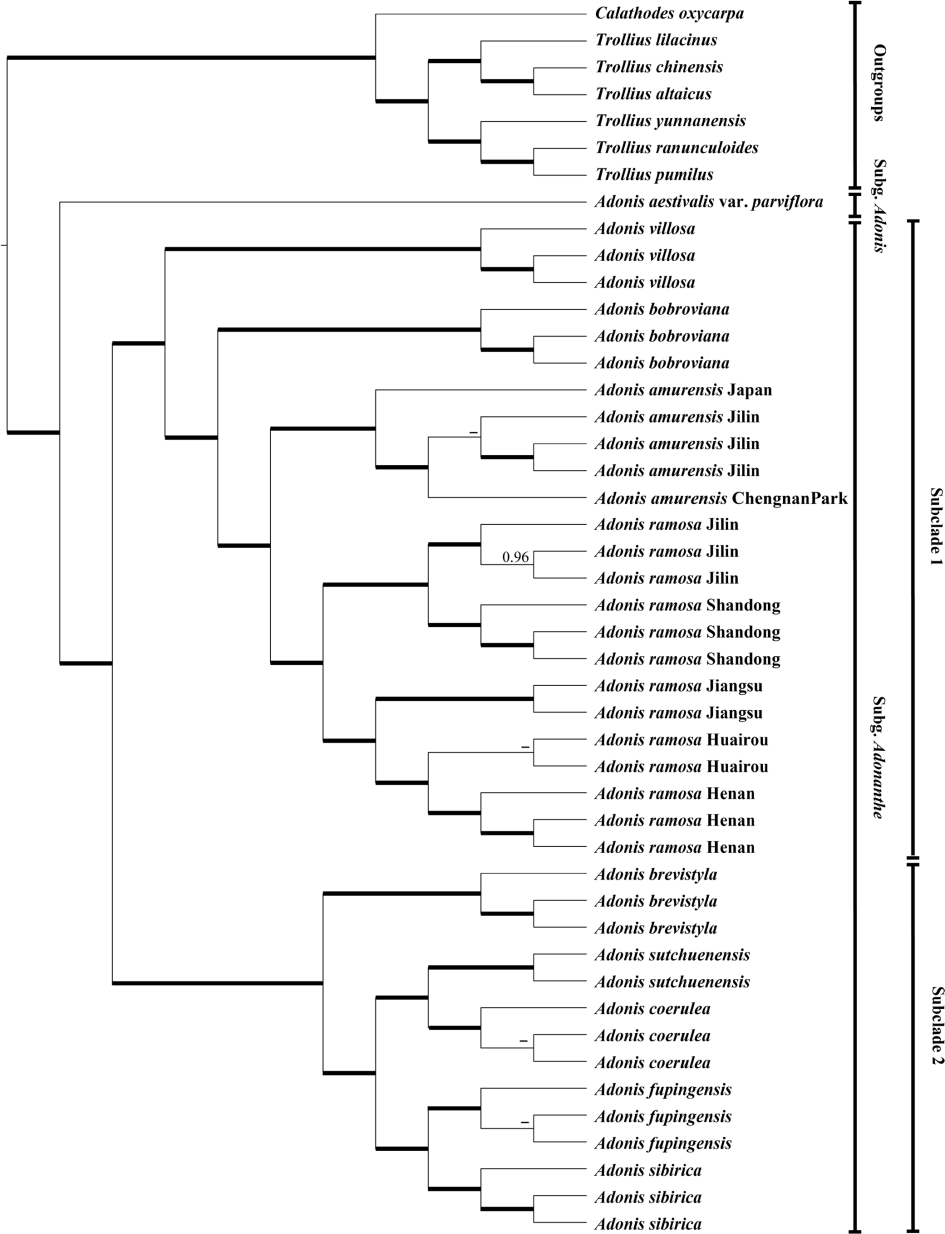
Coalescence‐based species tree topology inferred by SCOG1000 data set. Numbers at branches are local posterior probabilities (ASTRAL‐pp > 0.95), and bold branches mark ASTRAL‐pp equal to 1.00. ASTRAL‐pp values < 0.95 are shown as ‐.

**FIGURE 4:**
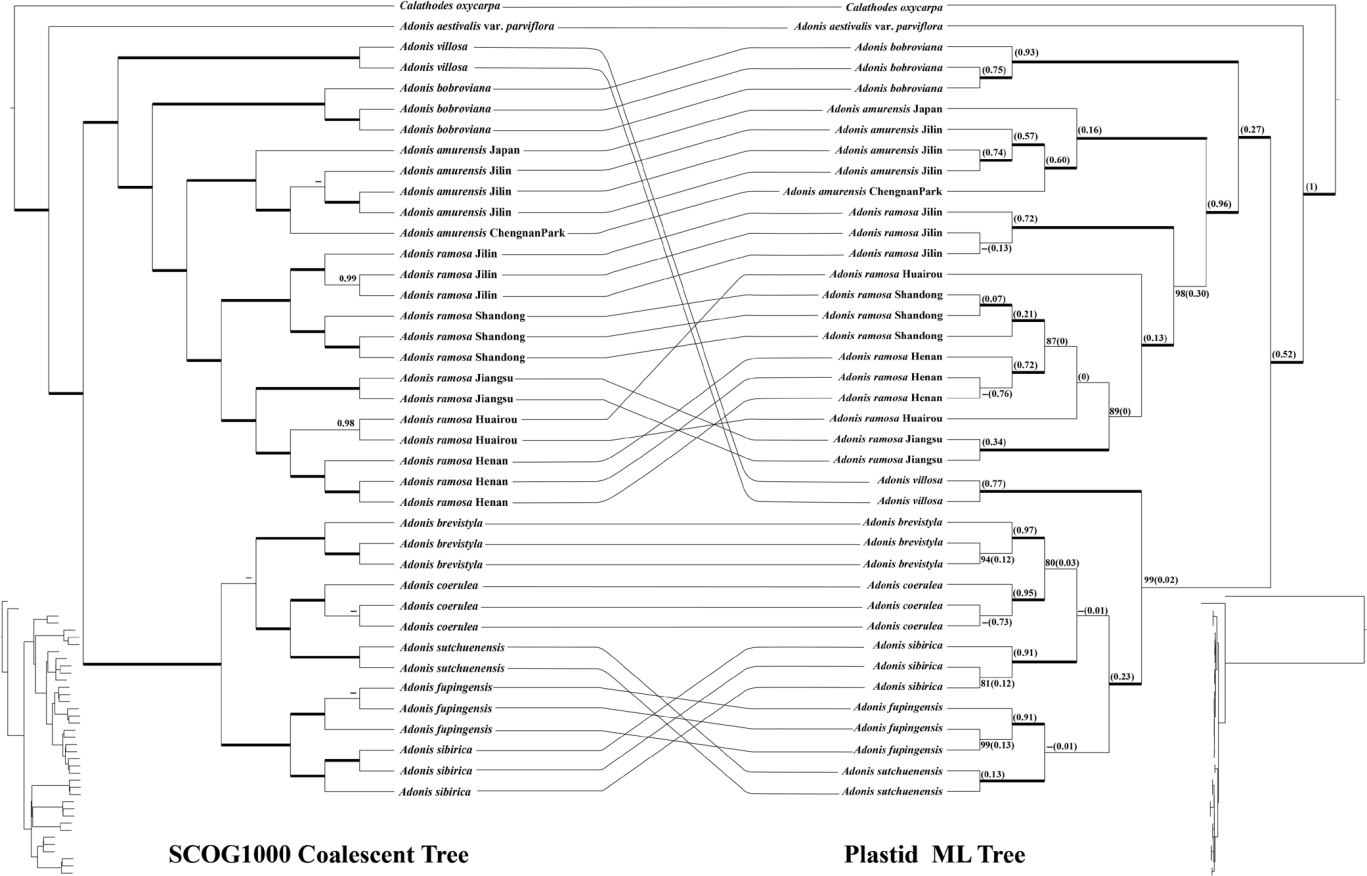
Discordance between the nuclear and plastid phylogenies. For brevity, only one outgroup was retained in the phylogenetic tree. Left: Coalescence‐based species tree topology inferred from SCOG1000 dataset. Local posterior probabilities (ASTRAL‐pp > 0.95) are shown at branches. ASTRAL‐pp values of 1.00 were denoted by bold branches. Right: The maximum likelihood (ML) tree inferred from the complete plastome sequences. Bootstrap values are indicated on the branches (outside brackets) and bold branches indicate ML bootstrap values of 100. Numbers in brackets show the contribution of incomplete lineage sorting to the conflicts between the nuclear and plastid gene trees based on the multispecies coalescent model. Trees with branch lengths of both datasets are shown left and right below.

Two transcriptome‐based datasets (SCOG1000 and SCOG2000) yielded generally congruent phylogenies in the coalescent‐based and concatenated analyses (Figure 3 and Figures [Link], [Link]). In both the concatenated and coalescent phylogenetic trees constructed from the SCOG1000 dataset, all but five nodes received 100% support values (Figure A3 and Figure 3, respectively). However, the SCOG2000 dataset exhibited slightly lower phylogenetic resolution than the SCOG1000 dataset, with 11 clades in the concatenation tree and 8 in the coalescent tree lacking 100% support (Figures A4 and A5). For the two nuclear datasets, the perennial subg. *Adonanthe* and the annual subg. *Adonis* also diverged first in the genus. Within the subg. *Adonis* clade, two major lineages (subclades) were also resolved. In contrast to the plastid tree (Figure 2), however, *A. villosa* was placed within subclade 1 in the phylogeny based on nuclear data (Figures 3 and 4, Figures [Link], [Link]). In subclade 2 of the four nuclear genome trees (Figure 3, Figures [Link], [Link]), the phylogenetic positions of *A. brevistyla* , *A. sutchuenensis*, and *A. coerulea* differed slightly from each other, showing uncertain relationships of these species. In contrast to the plastid phylogeny, the Fuping population formed a sister clade with *A. sibirica* in the nuclear gene tree. Within the *A. ramosa* clade in subclade 1, both plastid and nuclear phylogenetic trees showed that Jilin Tonghua population and Shandong Rushan population are sister groups within the species. Populations from Beijing, Henan, and Jiangsu provinces formed another cluster within the species, showing different evolutionary pathways of these eastern North China populations.

### 3.5 Gene Tree Discordance

**FIGURE 5:**
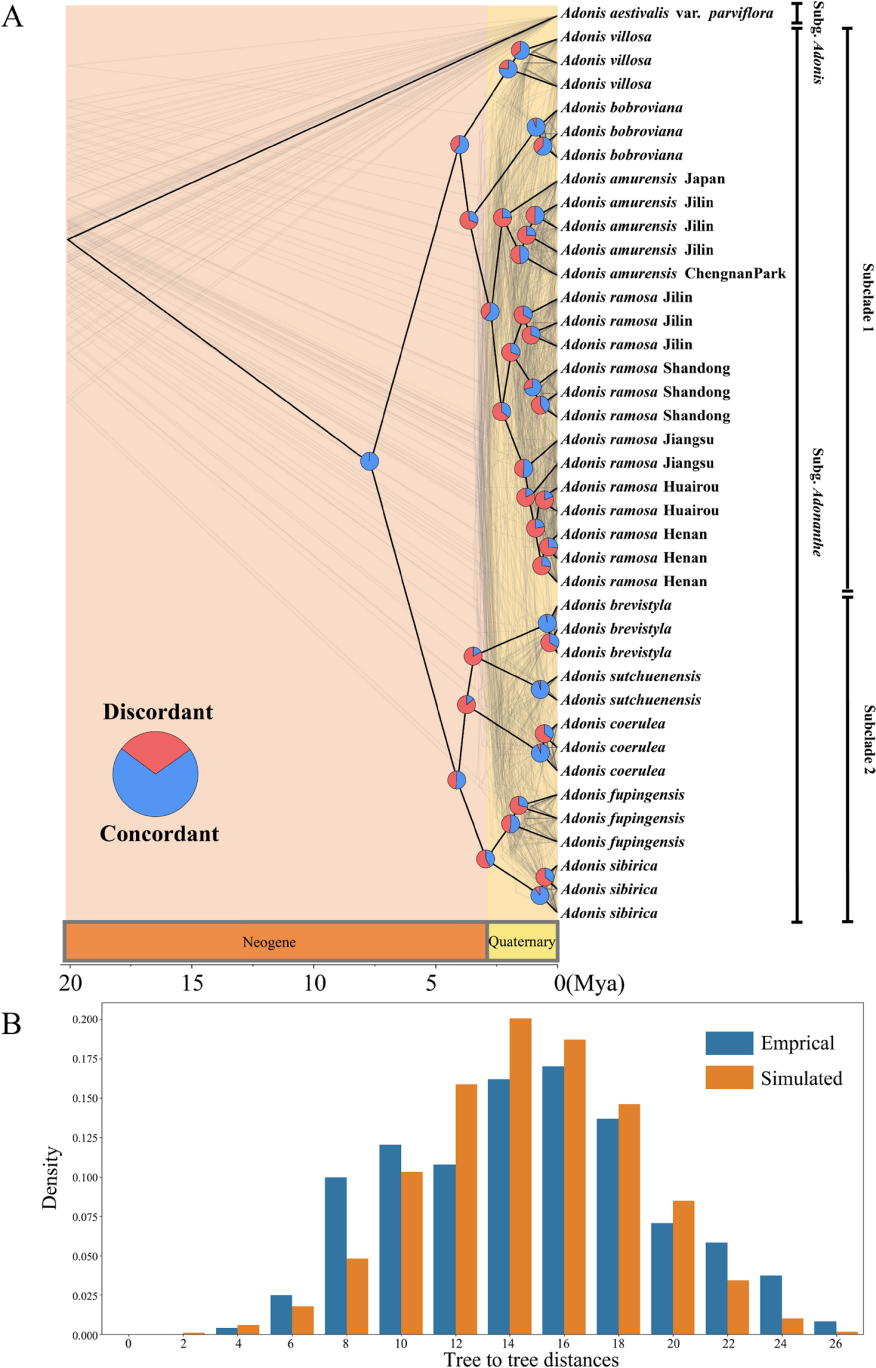
(A) Cloud tree showing discordance among nuclear genes. The ASTRAL species tree (based on trees from SCOG1000 dataset with average bootstrap value more than 60) is in heavy black lines. The gray‐colored trees (cloud tree) were sampled from 71 SCOGs (without missing taxa). Pie charts show the proportions of concordant and discordant topologies of gene trees comparing to the species tree. (B) Coalescent simulations of tree‐to‐tree distance distributions between the ASTRAL species tree and the 2242 empirical (orange boxes) gene trees (based on trees from SCOG1000 dataset with average bootstrap value more than 60) and those from the 10,000 simulation trees (blue boxes).

Using SCOG1000 (and average bootstrap value more than 60) dataset, high levels of gene tree discordances were detected within subg. *Adonanthe* (Figure 5A). Coalescent simulation analysis (Figure 5B) showed a similar pattern between empirical and simulated distance distributions, indicating that ILS alone can explain most of the gene tree conflicts. However, the contradiction between some nodes of the plastid and the nuclear species trees may not be explained by ILS (Figure 4). For instance, the significant cytonuclear discordance detected in *A. villosa* is suggestive of a putative hybrid origin. This hypothesis, however, necessitates verification via dedicated hybridization analysis.

### 3.6 Assessment of Hybridization

**FIGURE 6:**
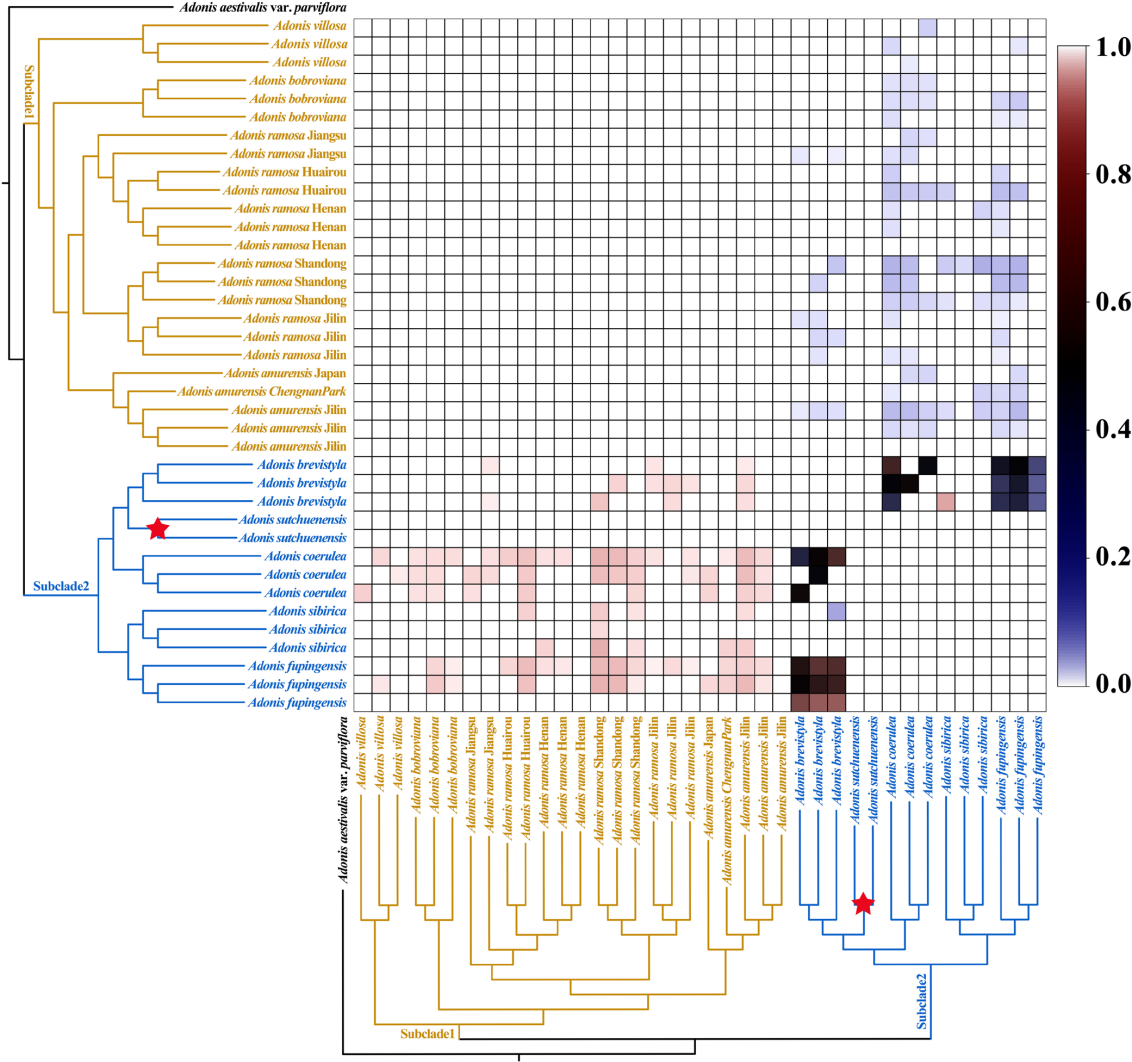
Inferred ancestral heatmap generated using “Node Mode” in VisualHyDe for the Most Recent Common Ancestor (MRCA) of *Adonis sutchuenensis* (marked by red star). The phylogenetic tree annotates the potential parents P1 (rows) and P2 (columns). Colors on heatmap represent significant inheritance probabilities (*γ*) from the row taxon (P1).

Our analysis, combining HyDe software with our custom VisualHyDe script, uncovered limited but clear evidence of interspecific hybridization events within the genus *Adonis* (Figure 6). The false‐positive filtering workflow confirmed the authenticity of these key ancestral signals, while successfully removing spurious signals caused by evolutionary rate heterogeneity. Complete results are available in Science Data Bank, doi: https://doi.org/10.57760/sciencedb.37469. The significant cytonuclear discordance in *A. villosa* was not accompanied by any detectable hybridization signal. Therefore, the conflicting phylogenetic histories of its nuclear and plastid genomes may have been attributed to evolutionary mechanisms, such as ILS, other than hybridization. Our analyses provide strong evidence for a hybrid origin of *A. sutchuenensis* (Figure 6). Both *A. brevistyla* (mean *γ* = 0.409 ± 0.062) and the Fuping population of *Adonis* (mean *γ* = 0.591 ± 0.062) were identified as genetic contributors to its genome. No clear evidence of hybridization was detected in the other sampled *Adonis* species in this study.

## 4 Discussion

### 4.1 Phylogenetic Position of *Adonis* in Eastern North China

Both the plastid and nrITS gene trees constructed using data from Ling et al. (2025) successfully resolved the annual subg. *Adonis* and the perennial subg. *Adonanthe* as distinct clades (Figures A1 and A2). However, the Sanger sequencing‐based trees generally suffered from insufficient statistical support, especially in subg. *Adonanthe*. Notably, multiple individuals of many species did not form monophyletic groups, a limitation mainly attributable to the limited phylogenetic information provided by Sanger sequencing data.

The Fuping population showed discordant placements in the plastid (some samples of *A. brevistyla* and *A. sutchuenensis*; Figure A1) and nrITS ( *A. sibirica* , *A. apennina*, and *A. mongolica* ; Figure A2) trees, without strong supports. These findings are largely consistent with the phylogenomic results obtained in the present study. While the present phylogenomic study did not include *A. apennina* in its sampling, the morphological synonymy of *A. apennina* and *A. sibirica* as established by W. T. Wang (1994a, 1994b) suggests that these two species should be treated as conspecific. The study by Ling et al. (2025) retained the two species names based on nomenclatural convention rather than taxonomic distinction. Similarly, the present phylogenomic study did not include *A. mongolica* . This species is narrowly distributed in the mountainous regions of western Mongolia and is extremely rare. It exhibits highly distinctive morphological features, including sessile cauline leaves, stems and leaves covered with short glandular hairs, white petals, and erect persistent styles (W. T. Wang 1994a, 1994b). These morphological traits are markedly distinct from those of *A. ramosa* , the Fuping population and any other *Adonis* species from China.

Our phylogenomic analyses, based on both plastid and nuclear genes, provide a robust phylogenetic framework for elucidating the taxonomic identities of the enigmatic *Adonis* populations in eastern North China. In agreement with earlier molecular studies, the primary divergence in our phylogeny separated subg. *Adonis* from subg. *Adonanthe* (Figures 2, 3, 4, 5, 6). The *Adonis* populations from eastern North China were resolved in two distinct subclades. The Fuping (Hebei) population grouped in subclade 2, in contrast to the populations from Beijing, Shandong, Henan, and Jiangsu, which all clustered within subclade 1. Populations of *Adonis* from Beijing, Shandong, Henan, and Jiangsu grouped together with *A. ramosa* of Jilin (NE China) and were sister to *A. amurensis* . The Fuping population from Hebei, however, was allied with either *A. sibirica* or *A. sutchuenensis*. This pattern highlights the critical importance of dense taxonomic sampling and genomic data in uncovering true species status and distributions, and challenging long‐held biogeographic assumptions (Li et al. 2023; Zhao et al. 2024).

Interestingly, the phylogenetic position of *A. villosa* (distributed in Central and Northern Asia) was incongruent between the plastid and nuclear genome trees, exhibiting conflicting positions in different subclades (Figures 2, 3, 4), which indicates a complex evolutionary history. Resolving the precise phylogenetic placement of this species will require future studies with expanded taxon sampling, particularly through the inclusion of sect. *Adonanthe* species outside China.

### 4.2 Cyto‐Nuclear Discordance Reveals a Complex Evolutionary History in Sect. *Adonanthe*

A key finding of our study is the prevalent nuclear and cyto‐nuclear discordance observed within the genus, underscoring a complex evolutionary history that cannot be fully captured by single‐genome or Sanger sequencing based analyses. The most striking case is *A. villosa* , which is robustly placed in subclade 2 of the plastome tree but within subclade 1 of the nuclear species tree (Figure 4). Such significant discordance often points to historical hybridization (Zhang et al. 2020; Rose et al. 2021). However, our analysis did not detect a clear signal of hybrid origin in *A. villosa* . The cytonuclear discordance observed in the phylogenetic placement of *A. villosa* could be attributed to biological processes other than hybridization, such as Incomplete Lineage Sorting (ILS). Alternatively, it may also stem from the limited sampling in this study, which focused on *Adonis* species in eastern North China and thus may have missed relevant hybridizing species from Central Asia.

It is noteworthy that while our phylogenic study revealed widespread phylogenetic discordance, both among nuclear genes and between nuclear and plastid genomes, within the genus *Adonis*, we detected only limited evidence for well‐defined hybridization events. Our coalescent simulations support that ILS can explain a substantial portion of the gene tree heterogeneity within the genus (Figure 5B). The deep coalescence of ancestral polymorphisms, likely facilitated by rapid diversification and/or small ancestral population sizes, can produce such strong and persistent phylogenetic conflicts (Morales‐Briones et al. 2021). This finding serves as a critical reminder that cyto‐nuclear discordance is not synonymous with hybridization and that rigorous testing of alternative hypotheses is essential (Rose et al. 2021).

Conversely, we identified a clear case of hybrid speciation in *A. sutchuenensis* (Figure 6). Our analysis, which incorporated stringent false‐positive filtering (He et al. 2025), confidently identified *A. brevistyla* and the Fuping population (*A. fupingensis*) as its parental donors. This also explains why it was difficult to confidently identify the closest relatives of the Fuping population within Subclade 2. This result not only elucidates the origin of a distinct species but also demonstrates the power of phylogenomic datasets and sophisticated analytical tools to detect ancient hybridization events that have shaped the genus's diversity. The fact that such events appear to be limited within the genus suggests that reproductive barriers are generally strong, yet hybridization can occasionally act as a creative evolutionary force in *Adonis*.

### 4.3 Implications for the Infrageneric Classification of *Adonis*

Our genome‐scale phylogeny largely corroborates the major outlines of W. T. Wang's (1994a, 1994b) classification, strongly supporting the monophyly of the annual subg. *Adonis* and the perennial subg. *Adonanthe*. This taxonomic delineation is consistent with prior molecular phylogenetic studies (Ling et al. 2025). This consistency between classical morphology and phylogenetic studies validates the utility of life history and key morphological traits as indicators of deep evolutionary splits within the genus. However, molecular phylogenetic evidence provides limited support for the current sectional classification (sensu W. T. Wang 1994a, 1994b) of *Adonis*. The species‐rich sect. *Adonanthe* within subg. *Adonanthe* (W. T. Wang 1994a, 1994b) has been confirmed as monophyletic in molecular phylogenetic analyses (Ling et al. 2025). The defining characteristics of this section are as follows: basal and lower cauline leaves are reduced to pale brown; middle and upper cauline leaves are well‐developed, 2‐ to 4‐times pinnatisect, with ovate to triangular blades; and the pubescent achenes have a strongly convex, rounded‐truncate dorsal apex, often with wrinkled or reticulate sides, and a short, strongly recurved persistent style 0.4–1 mm long. Most *Adonis* species distributed in China belong to this section. W. T. Wang (1994a, 1994b) further divided sect. *Adonanthe* into four series based on leaf morphology, pollen characteristics, and persistent style morphology. However, these series are not supported by molecular phylogenetic studies (Ling et al. 2025).

Our results indicate that the species of sect. *Adonanthe* distributed in China are resolved into two subclades. With the exception of *A. villosa* , both subclades demonstrated stable species compositions. All *Adonis* populations from eastern North China, with the exception of the Fuping (Hebei) population, clustered with *A. ramosa* within subclade 1 and were sister to *A. amurensis* . In contrast, the Fuping population was consistently placed within subclade 2 in both the plastome and nuclear phylogenetic trees. Consequently, these results support that most *Adonis* populations in eastern North China belong to *A. ramosa* , and reject the hypotheses that the Fuping population represents *A. amurensis* (J. W. J. W. Wang 1986), a subspecies of *A. ramosa* (W. T. Wang 1994a, 1994b), or a synonym of *A. ramosa* (Fu and Robinson 2001). Instead, our results confirm that the Fuping population represents a unique evolutionary lineage, necessitating its recognition at the species level as *A. fupingensis*.

Although our results indicate that *A. fupingensis* is phylogenetically close to *A. sibirica* , *A. mongolica* , or *A. sutchuenensis*, it is morphologically distinct from all of them. *Adonis fupingensis* is characterized by its relatively short stature and the presence of a distinct petiole, which clearly differentiates it from *A. sibirica* (or *A. apennina* according to W. T. Wang 1994a, 1994b), a species distributed in Xinjiang Province and adjacent areas. As mentioned above, *A. mongolica* is distinctly different from other species from China due to its white flowers, erect persistent styles, and plants covered with short glandular hairs. *Adonis sutchuenensis*, however, exhibits broader leaf blades with wider lobes and is further characterized by its larger flowers and more elongated petals compared to *A. fupingensis*. In terms of overall appearance, *A. fupingensis* indeed more closely resembles *A. ramosa* from China. Their shared morphological features include petiolate and glabrous basal leaves, a calyx half the length of the petals, and yellow petals. It is no wonder that some taxonomists have identified *A. fupingensis* as *A. ramosa* .

**FIGURE 7:**
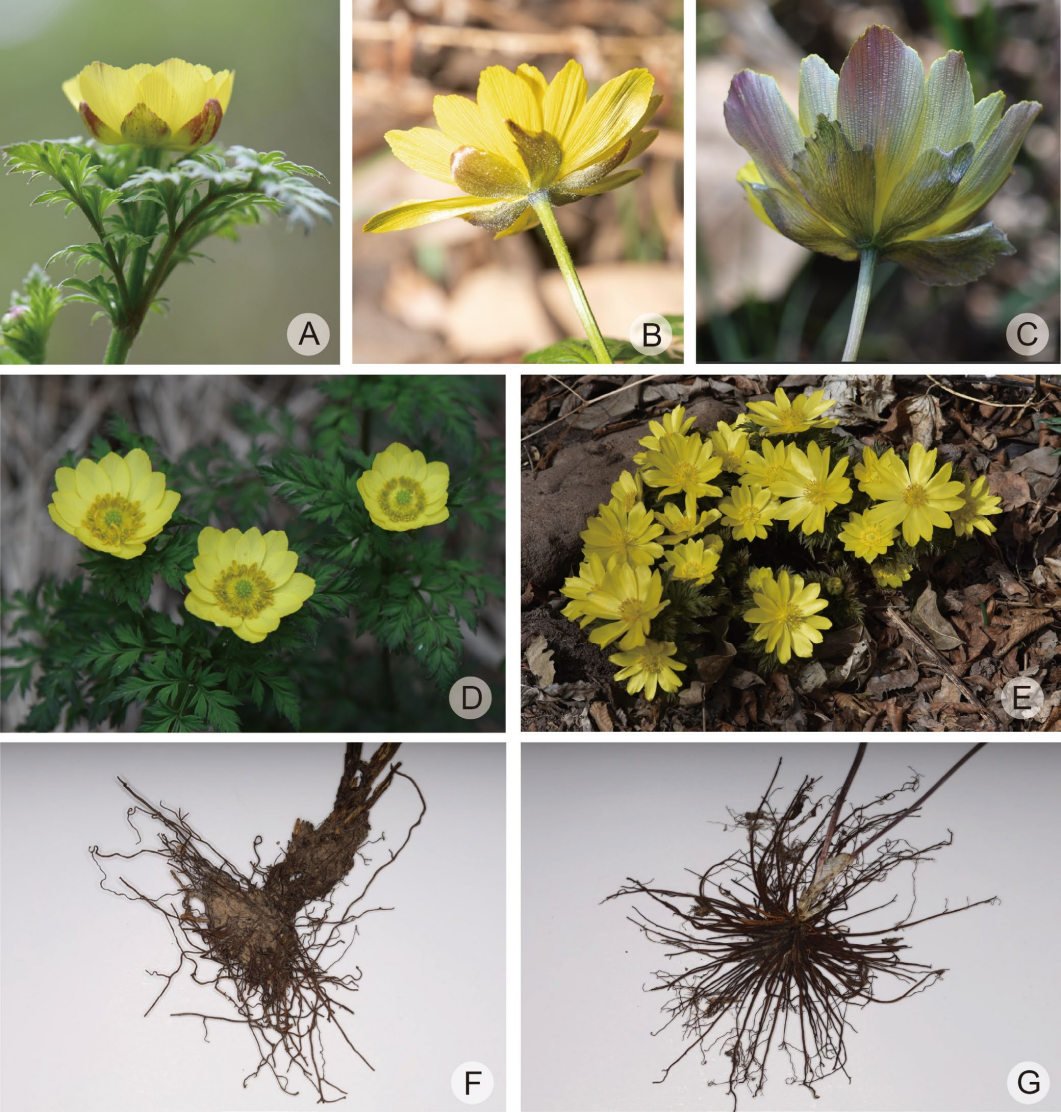
Photographs of *Adonis fupingensis* and *A. ramosa* from China. (A) *A. fupingensis*; (B) *A. ramosa* from Jiangsu Province; (C) *A. ramosa from* Shandong Province. (A–C) illustrate the differences in the sepals of *A. fupingensis* and *A. ramosa* , respectively. (D) *A. fupingensis*; (E) *A. ramosa* from Henan Province. (D and E) illustrate the differences in petal shape between *A. fupingensis* and *A. ramosa* . (F) Roots of *A. fupingensis*; (G) Roots of *A. ramosa* . (F and G) illustrate the differences in the roots between *A. fupingensis* and *A. ramosa* . (photoed by Lei Xie, Wen‐He Li, and Jia‐Min Xiao).

However, our field investigations and herbarium studies have revealed consistent but subtle morphological differences between *A. fupingensis* and *A. ramosa* that are easily overlooked (Figure 7). First, *A. fupingensis* has sepals that are often ovate, purple early in anthesis, and have entire margins (Figure 7A), while those of *A. ramosa* are typically lanceolate to narrow‐ovate, yellowish‐green or faintly purplish, and often lobed or toothed apically (Figure 7B,C). Second, *A. fupingensis* possesses ovate and rather broad petals (Figure 7D), whereas those of *A. ramosa* exhibit a range of narrower forms, encompassing linear‐lanceolate, lanceolate, to narrow‐ovate shapes, all of which are more elongated (Figure 7E). Third, and most notably, a distinct difference lies in their underground parts, which is often the most overlooked. *Adonis fupingensis* has relatively a few fibrous roots (Figure 7F), which are slender and highly branched. In contrast, *A. ramosa* possesses numerous, thick, fleshy roots with considerably fewer branches (Figure 7G). The root system of *A. fupingensis* is more similar to that of *A. brevistyla* and *A. coerulea* in this regard. Finally, it is important to note that there is a distinct difference in flowering phenology between the two species. *Adonis ramosa* blooms in early spring, with populations in eastern North China flowering from February to mid‐April. In contrast, *A. fupingensis* flowers in early summer, around early to mid‐May. The integrated evidence from morphology, phenology, and phylogenetic genomics leads us to propose that the *Adonis* population from Hebei Fuping represents a distinct species.

Furthermore, the phylogenetic uncertainty among *A. brevistyla* , *A. sutchuenensis*, and *A. coerulea* in the nuclear trees (Figure 3), coupled with the evidence of hybridization for *A. sutchuenensis*, indicates that evolutionary relationships within subclade 2 are particularly intricate. These complexities, likely stemming from a combination of ILS and reticulate evolution, pose a challenge for establishing a strictly bifurcating infrageneric classification for this group. Our study thus aligns with a growing body of literature advocating for a phylogenetic framework that acknowledges and incorporates these non‐tree‐like evolutionary processes (Zhang et al. 2020; Colli‐Silva et al. 2025). Future taxonomic revisions of *Adonis* may benefit from an integrative approach that reflects this complex history.

## 5 Taxonomic Treatment

**Adonis fupingensis** (W. T. Wang) Wen He Li & L. Xie, **stat. et sp. nov**.

**Basionym**: *Adonis ramosa* subsp. *fupingensis* W. T. Wang, in Acta Phytotax. Sin. 32: 472, 1994.

**Type**: China, Hebei, Fuping, 1500–1600 m, *Chanet A390* [holotype: TIE!].

**Additional specimen cited**: China, Hebei, Fuping Co., Liaodaobei, alt. 1950 m, 17 May, 2023, *L. Xie et al. 2023051706* (BJFC); the same locality, 5 July, 2025, *L. Xie* and *Wen‐He Li 2025070502* (BJFC).

**Ecology, distribution, and status**: This species was known from only one locality in western Hebei Province. In Liaodaobei, Fuping Co., individuals of this species grow under larch forests near the ridge‐line or thrive in grassy slopes along the mountain ridge. The area we discovered is rather small, including a few dozen individuals. This species' habitat is highly vulnerable to anthropogenic disturbances, which may lead to population degradation or even local extinction. According to the IUCN red list categories and criteria (IUCN Standard and Petitions Committee 2022), *Adonis fupingensis* should be categorized as critically endangered (CR).

## 6 Conclusions

This integrated phylogenomic study successfully resolves the long‐standing taxonomic enigma surrounding *Adonis* populations in eastern North China. We delineate *A. fupingensis* as a distinct species endemic to the Taihang Mountains and clarify the broader distribution of *A. ramosa* in the region. Beyond taxonomy, our analyses uncovered a complex evolutionary history characterized by widespread gene tree discordance. We demonstrate that while incomplete lineage sorting has been a major factor in shaping these patterns, ancient hybridization has also played a definitive role in the origin of at least one species, *A. sutchuenensis*. Our study underscores the critical importance of employing both cytoplasmic and nuclear genomic datasets to distinguish between alternative evolutionary processes such as ILS and hybridization. By doing so, this work not only provides a robust phylogenetic foundation for future biogeographic and conservation studies in *Adonis* but also contributes to the growing consensus that the evolutionary histories of many plant groups are best represented as networks rather than simple trees. Despite significant prior taxonomic work on groups like Ranunculaceae, our findings emphasize that persistent controversies can often only be resolved through the application of phylogenomic methodologies and the integration of multidisciplinary evidence.

## Author Contributions

**Wen‐He Li:** data curation (equal), formal analysis (equal), investigation (lead), writing – review and editing (equal). **Ming‐Yang Li:** investigation (lead), writing – review and editing (equal). **Bo‐Wen Liu:** investigation (lead), writing – original draft (supporting). **Jian He:** writing – review and editing (lead). **Jia‐Min Xiao:** investigation (equal), resources (equal). **Le‐Le Lin:** investigation (supporting). **Ce Shang:** investigation (supporting). **Huanyu Wu:** investigation (supporting). **Shuang Qin:** investigation (supporting). **Zi‐Yi Li:** investigation (supporting). **Jin Cheng:** project administration (lead), supervision (lead). **Lei Xie:** conceptualization (equal), funding acquisition (lead).

## Funding

This research was funded by the Discipline Crossing Foundation of School of Ecology and Nature Conservation, Beijing Forestry University (grant number: BH2025‐JX‐03); the National Natural Science Foundation of China (grant number: 31670207).

## Conflicts of Interest

The authors declare no conflicts of interest.

## Supporting information


**Figure A1:** Maximum likelihood phylogram constructed using eight plastid regions (*mat*K, *rbc*L, *atp*B‐*rbc*L, *rps*16, *trn*G, *trn*H‐*psb*A, *trn*L‐F, *trn*S‐*trn*G). The samples include all samples from Ling et al. (2025) and all *Adonis* samples from this study. Bootstrap values (> 60) and Bayesian posterior probabilities (> 0.95) are indicated on the branches. Internal branches, which are fully supported by ML bootstrap and Bayesian analyses, are in bold.

**Figure A2:** Maximum likelihood phylogram constructed using the nrITS region. The samples include all samples from Ling et al. (2025) and all *Adonis* samples from this study. Bootstrap values (> 60) and Bayesian posterior probabilities (> 0.95) are indicated on the branches. Internal branches, which are fully supported by ML bootstrap and Bayesian analyses, are in bold.

**Figure A3:** Concatenate‐based tree topology inferred by SCOG1000 data set. Its phylogram is shown left below. ML bootstrap values (> 60) are shown at each node. Internal branches, which are fully supported by ML bootstrap analysis, are in bold.

**Figure A4:** Concatenate‐based tree topology inferred by SCOG2000 data set. Its phylogram is shown left below. ML bootstrap values (> 60) are shown at each node. Internal branches, which are fully supported by ML bootstrap analysis, are in bold.

**Figure A5:** Coalescence‐based species tree topology inferred by SCOG2000 data set. Coalescent tree with branch‐length is shown left below. Numbers at branches are local posterior probabilities (ASTRAL‐pp > 0.95), and bold branches mark ASTRAL‐pp equal to 1.00. ASTRAL‐pp values < 0.95 are shown as ‐.

**Table A1:** Plastid genome features and GenBank accession numbers of *Adonis* species in this study.

**Table A2:** Features and online accession numbers of RNA‐Seq data in this study.

## Data Availability

The DNA sequences generated in the present study have been deposited in the National Center for Biotechnology Information (NCBI) database. The accession numbers and the information on the voucher specimens are available in Tables A1 and A2. The results of hybridization detection are openly available in the Science Data Bank at doi: https://doi.org/10.57760/sciencedb.37469. The voucher specimens have been deposited at BJFC.
