# Supplementary figures and images for: Resolving the Taxonomic Enigma of Adonis in Eastern North China: An Integrated Phylogenomic Approach
Source: Ecol Evol. 2026 Mar 19;16(3):e73266. doi: 10.1002/ece3.73266 (PMC13093438; doi:10.1002/ece3.73266)

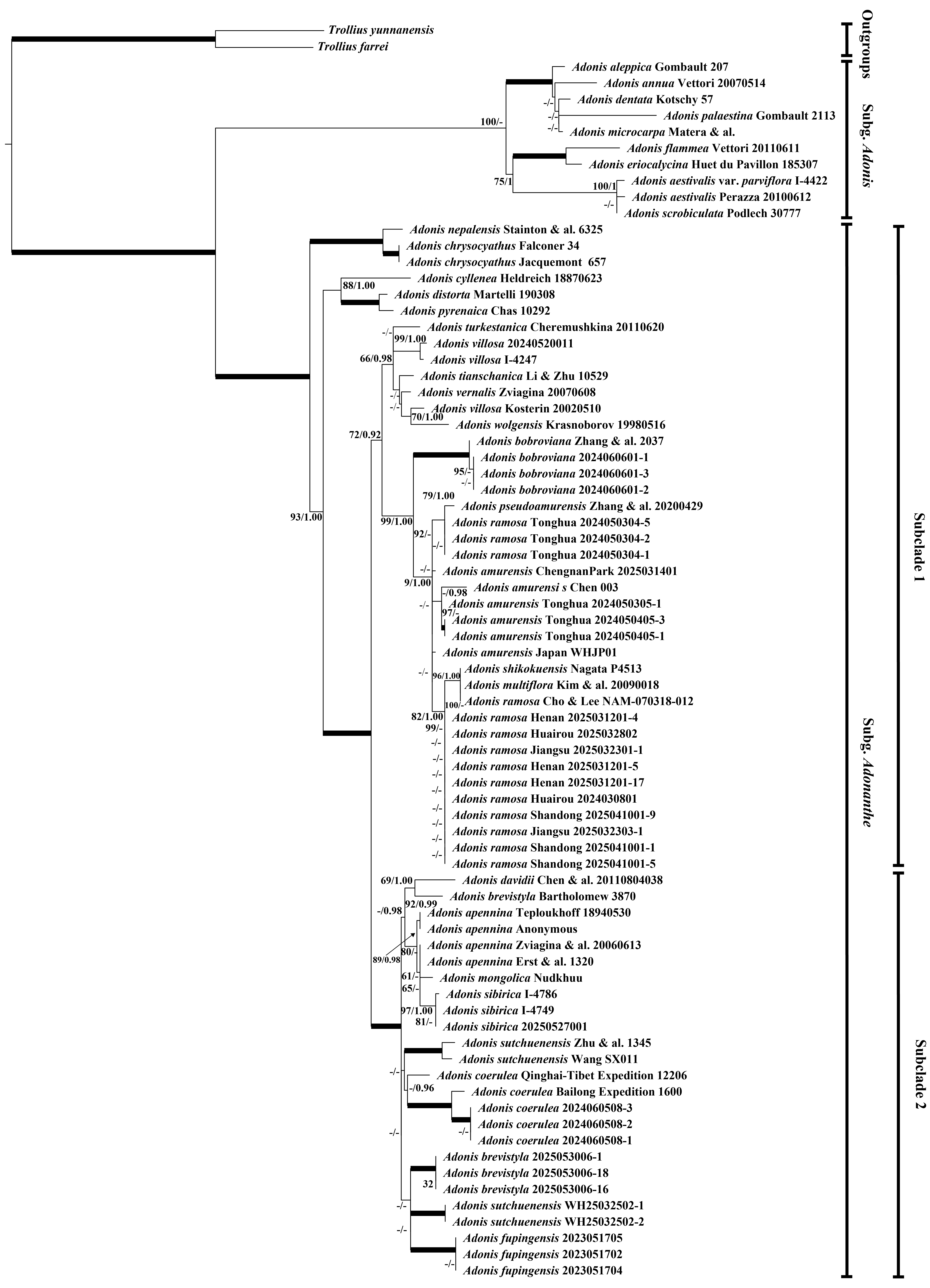

Supplement: Supplementary file 1 — Figure A1: Maximum likelihood phylogram constructed using eight plastid regions (matK, rbcL, atpB‐rbcL, rps16, trnG, trnH‐psbA, trnL‐F, trnS‐trnG). The samples include all samples from Ling et al. (2025) and all Adonis samples from this study. Bootstrap values (> 60) and Bayesian posterior probabilities (> 0.95) are indicated on the branches. Internal branches, which are fully supported by ML bootstrap and Bayesian analyses, are in bold. [file ECE3-16-e73266-s004.jpg]

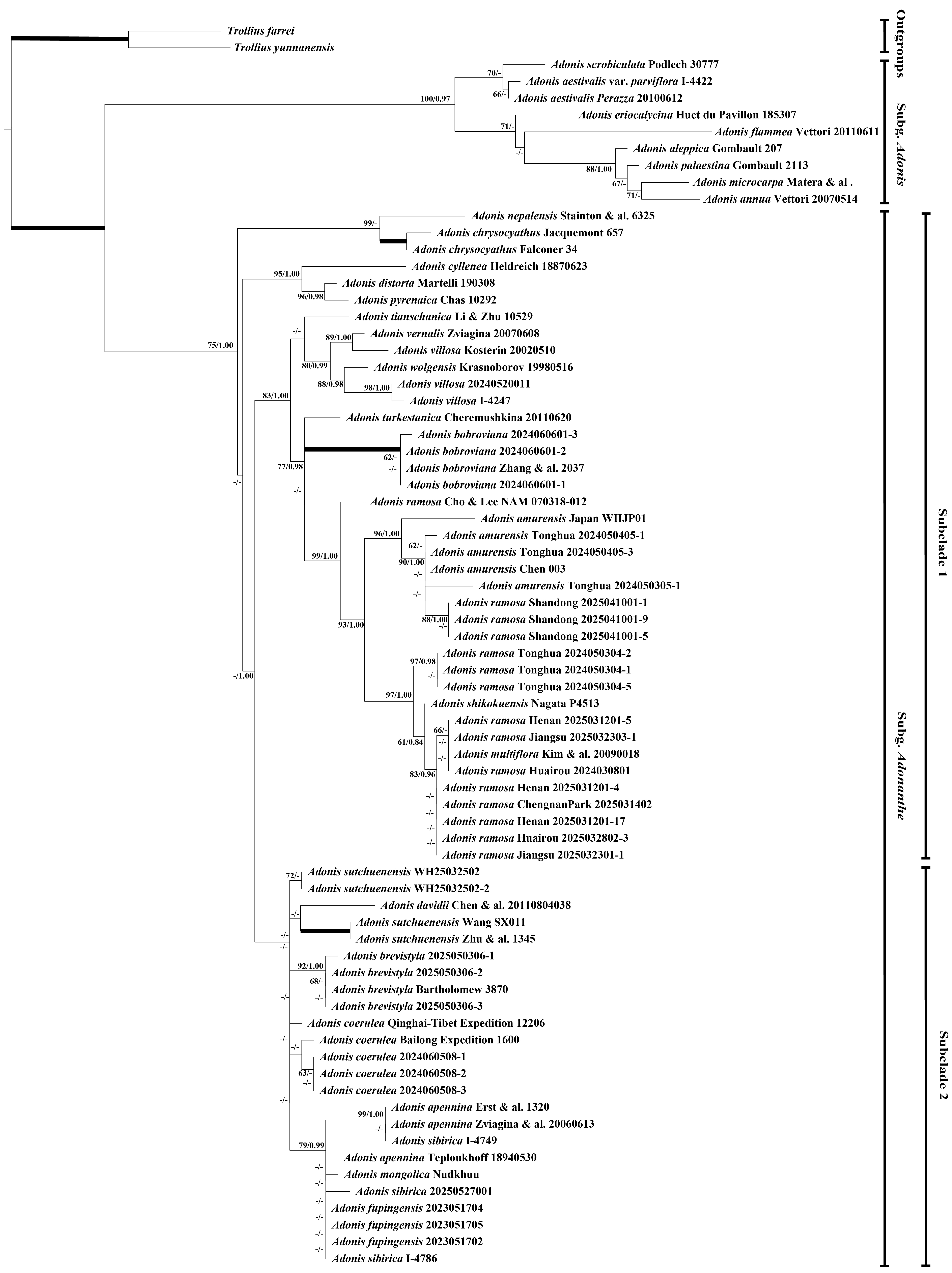

Supplement: Supplementary file 2 — Figure A2: Maximum likelihood phylogram constructed using the nrITS region. The samples include all samples from Ling et al. (2025) and all Adonis samples from this study. Bootstrap values (> 60) and Bayesian posterior probabilities (> 0.95) are indicated on the branches. Internal branches, which are fully supported by ML bootstrap and Bayesian analyses, are in bold. [file ECE3-16-e73266-s006.jpg]

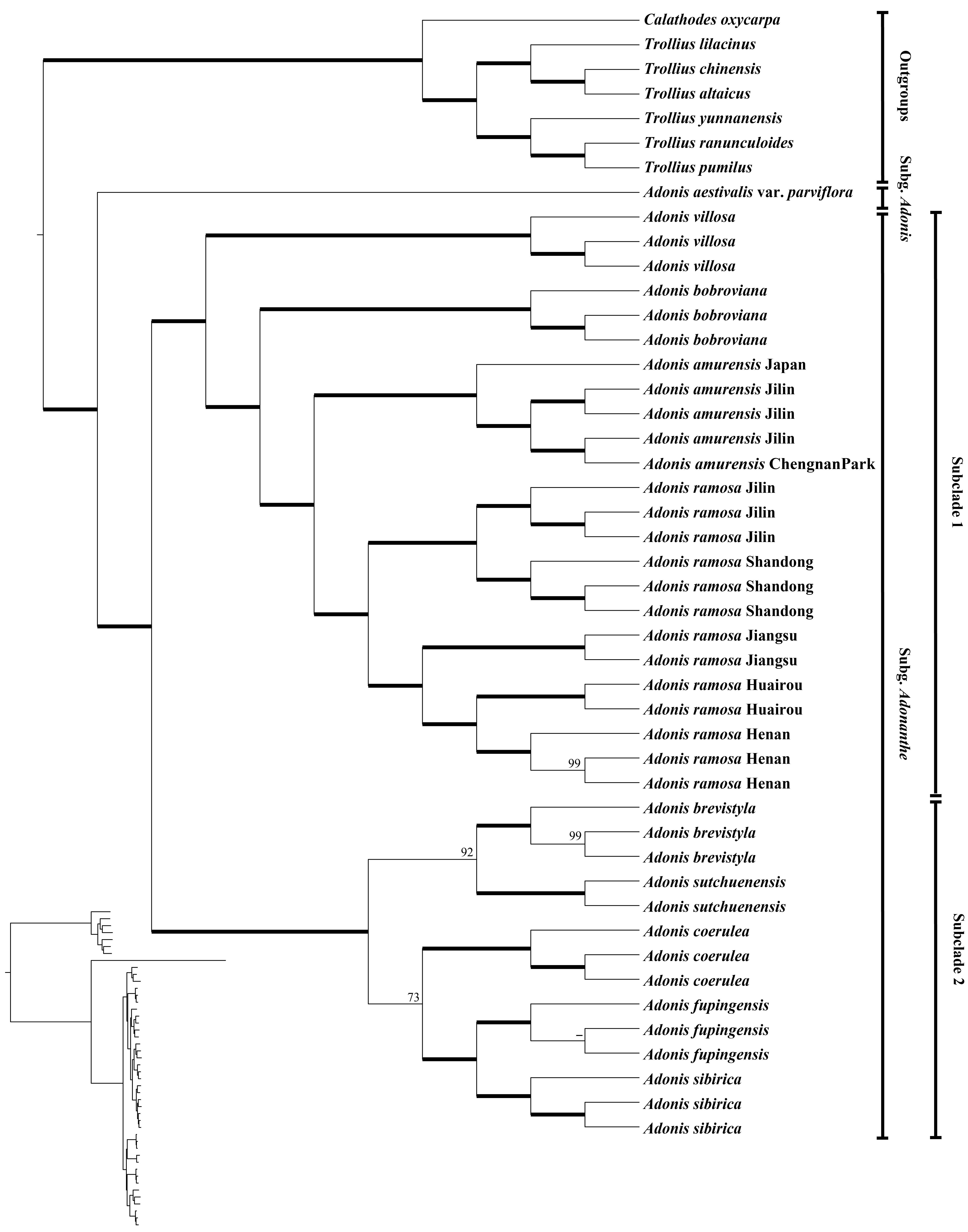

Supplement: Supplementary file 3 — Figure A3: Concatenate‐based tree topology inferred by SCOG1000 data set. Its phylogram is shown left below. ML bootstrap values (> 60) are shown at each node. Internal branches, which are fully supported by ML bootstrap analysis, are in bold. [file ECE3-16-e73266-s005.jpg]

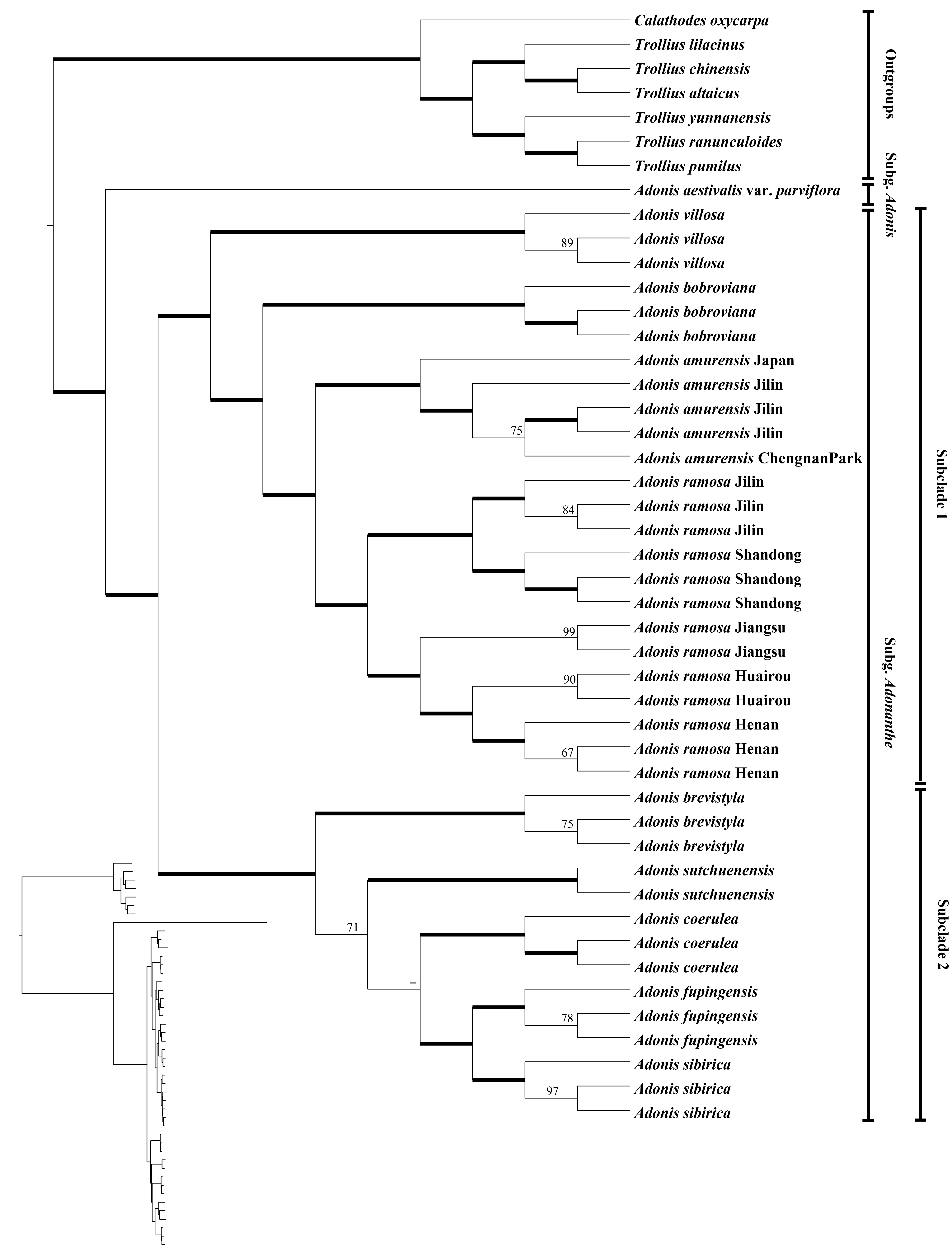

Supplement: Supplementary file 4 — Figure A4: Concatenate‐based tree topology inferred by SCOG2000 data set. Its phylogram is shown left below. ML bootstrap values (> 60) are shown at each node. Internal branches, which are fully supported by ML bootstrap analysis, are in bold. [file ECE3-16-e73266-s007.jpg]

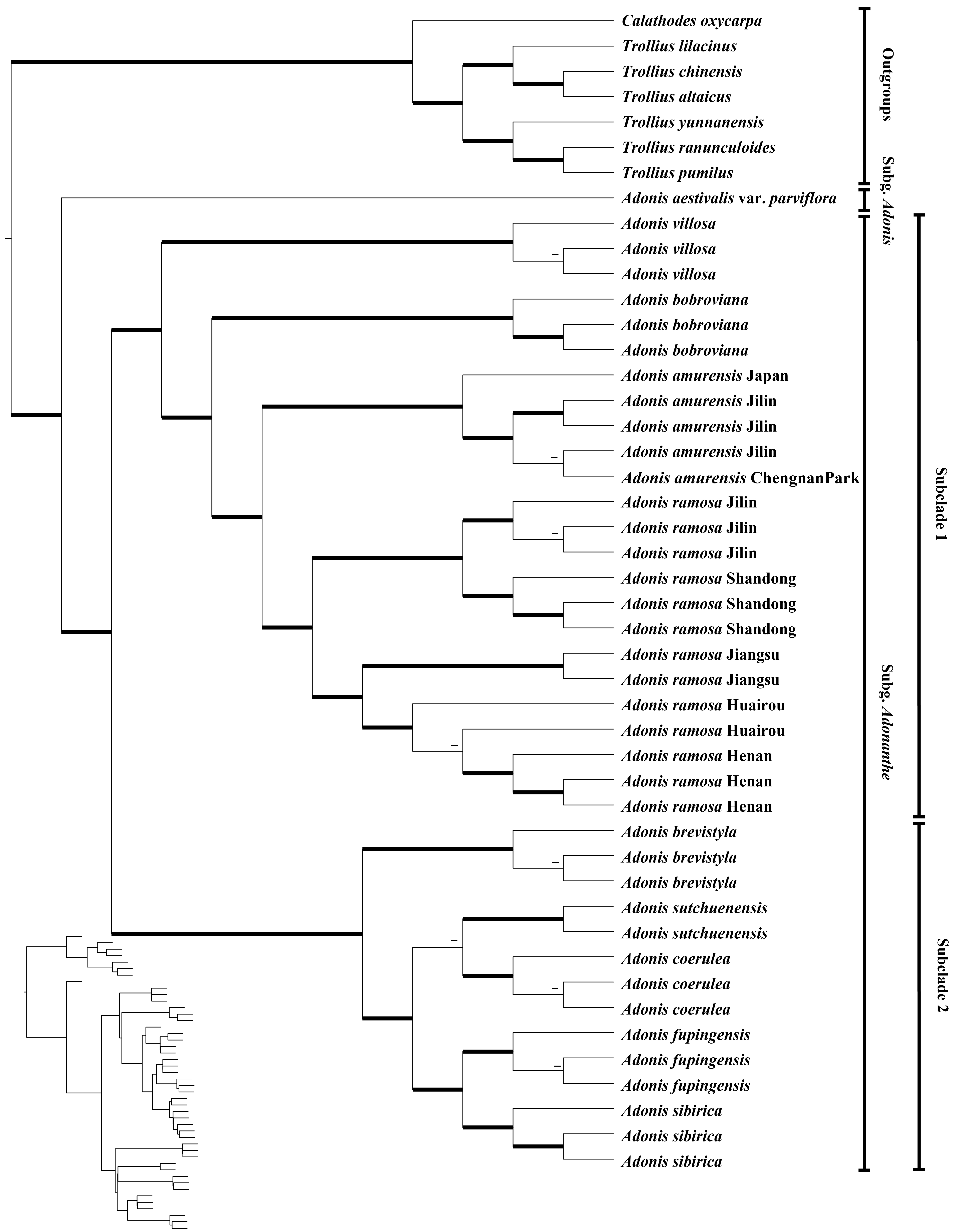

Supplement: Supplementary file 5 — Figure A5: Coalescence‐based species tree topology inferred by SCOG2000 data set. Coalescent tree with branch‐length is shown left below. Numbers at branches are local posterior probabilities (ASTRAL‐pp > 0.95), and bold branches mark ASTRAL‐pp equal to 1.00. ASTRAL‐pp values < 0.95 are shown as ‐. [file ECE3-16-e73266-s003.jpg]
